# (NH_4_)_2_[Tc_2_O_2_(S_2_O_7_)_4_]: An Unexpected Technetium(V)‐Sulfate From the Reaction of (NH_4_)(TcO_4_) With SO_3_

**DOI:** 10.1002/chem.202502493

**Published:** 2025-11-30

**Authors:** Stefan Sutorius, Julian Heinen, David van Gerven, Jörn Bruns, Erik Strub, Mathias S. Wickleder

**Affiliations:** ^1^ Institute of Inorganic Chemistry University of Cologne Cologne Germany; ^2^ Division of Nuclear Chemistry University of Cologne Cologne Germany; ^3^ Institute of Chemistry Technical University of Berlin Berlin Germany

**Keywords:** pertechnetate, rhenium, sufates, sulfur trioxide, super acid

## Abstract

The reaction of ammonium pertechnetate, (NH_4_)(TcO_4_), with SO_3_ leads to crystals of (NH_4_)_2_[Tc_2_O_2_(S_2_O_7_)_4_] (monoclinic, *P*2_1_/*c*, *Z* = 2, *a* = 775.67(5), *b* = 1339.55(8), *c* = 1233.32(8) pm, *β =* 100.566(2)°). The compound contains the dimeric [Tc_2_O_2_(S_2_O_7_)_4_]^2−^ anion with the Tc atom connected by disulfate anions. It is astonishing that even under the oxidizing reaction conditions, reduction of the Tc(VII) starting material occurs. Contrastingly, an analogous reaction of (NH_4_)(ReO_4_) with SO_3_ gives the Re(VII) sulfate (NH_4_)[ReO_2_(S_2_O_7_)_2_] (orthorhombic, *Pca*2_1_, *Z* = 8, *a* = 1842.7(1), *b* = 847.03(5), *c* = 1663.8(1) pm). The compounds have been further studied by vibrational spectroscopy and quantum mechanical calculations.

## Graphical abstract

The reaction of the radioactive pertechnetate (^99^TcO_4_)^−^ with the oxidizer SO_3_ leads to blue crystals of (NH_4_)_2_[Tc_2_O_2_(S_2_O_7_)_4_]. The complex dimeric [Tc_2_O_2_(S_2_O_7_)_4_]^2−^ anion exhibits the technetium in the oxidation state V, showing that reduction occurs during the reaction.

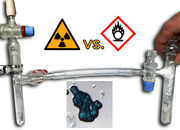

## 1 Introduction

The technetium isotope ^99^Tc is a β^‒^ emitter with a half‐life of 210 000 years. The metal is a fission product of nuclear power plants and occurs in spent fuel in its elemental state. The treatment of spent fuel is done by the so‐called PUREX process, which is designed to separate uranium and plutonium. PUREX stands originally for Plutonium‐Uranium Recovery by Extraction [1, 2], but Shafer et al. pointed out that *R* might also stand for *reduction* due to the importance of the plutonium reduction during the process [3]. In this process, uranium is oxidized by nitric acid to the uranyl cation, (UO_2_)^2+^, which is transferred as the tributylphosphate (TBP) complex of its nitrate into an organic solvent phase (dodecane). During the HNO_3_ treatment, the technetium metal present in the spent fuel is oxidized to the pertechnetate ion, (TcO_4_)^−^. In this form, it partially enters the organic phase along with uranium, possibly in the form of the compound UO_2_(NO_3_)(TcO_4_)(TBP)_2_ [4, 5], or, as discussed more recently, even as the neutral complex [HTcO_4_(H_2_O)_8_(TBP)_4_] [6]. However, part of the technetium remains in the aqueous phase, most likely as Tc(IV) species. In order to understand the redox chemistry of technetium in strong Brønsted acids like HNO_3_, several investigations have been reported, aiming at the speciation of technetium in these media [7, 8, 9, 10, 11]. The investigations included also sulfuric acid, H_2_SO_4_ [12], and its derivate trifluoromethanesulfonic acid (“triflic acid”), CF_3_SO_3_H [13, 14]. Several complexes of Tc(VII) and Tc(V) have been shown to exist in these acids, however, structural proofs are missing. Throughout the last decades, we have gained strong experience in performing reactions in strong Brønsted acids and their anhydrides under harsh conditions, and quite a number of unexpected compounds resulted from these reactions. The blue paramagnetic disulfate Pd(S_2_O_7_), obtained from elemental palladium and neat SO_3_ may serve as a striking example [15], as well as the oxidation of elemental platinum by H_2_SO_4_ at 300 °C under formation of the Pt(III) species Pt_2_(SO_4_)_2_(HSO_4_)_2_ [16]. The results show, that SO_3_ and even H_2_SO_4_ are strong oxidizers, depending on the reaction conditions. This can be attributed to the high oxidation state of sulfur in these compounds and the formation of stable SO_2_ by reduction. Based on our fundamental experience in the chemistry of highly concentrated oxo‐acids, we recently started to evaluate structures of oxoanionic technetium compounds. As a first result, we could show, that in concentrated triflic acid ammonium pertechnetate, (NH_4_)(TcO_4_), reacts under formation of (TcO_3_)(CF_3_SO_3_) [16]. Interestingly, increasing reaction time leads to another species, namely (NH_4_)_2_[TcO(CF_3_SO_3_)_5_](CF_3_SO_3_H), containing a complex anion of Tc(V) [17, 18]. In the above‐mentioned speciation studies, the presence of pentavalent technetium has also been shown, however, as the reduction source, the presence of α‐radiation was assumed [12]. In principle, the observed reduction of technetium follows a trend, that the elements of the second and third row of the transition metals show increasing differences in their behavior within the respective periods. For example, while Zr and Hf, as well as Nb and Hf are very similar, the metals Rh and Ir, Ru and Os, show already severe differences, especially with respect to the stability of high oxidation states. Thus, Tc is of special importance, as it is located in the center of the transition metal block. In continuation of our work, we have now investigated how (NH_4_)(TcO_4_) behaves in a reaction with SO_3_, the anhydride of sulfuric acid. The strong oxidation power of SO_3_ should prevent reduction to Tc(V) during the reaction. Surprisingly, we were able to isolate the dimeric complex [Tc_2_O_2_(S_2_O_7_)_4_]^2−^ as the respective ammonium salt. Thus, also under the strong oxidizing condition reduction of Tc(VII) takes place. Contrastingly, the reaction of (NH_4_)(ReO_4_) with SO_3_ leads not to a reduction of the perrhenate anion but leads to the Re(VII) compound (NH_4_)[ReO_2_(S_2_O_7_)_2_], which will be also presented here.

## 2 Results and Discussion

**FIGURE 1:**
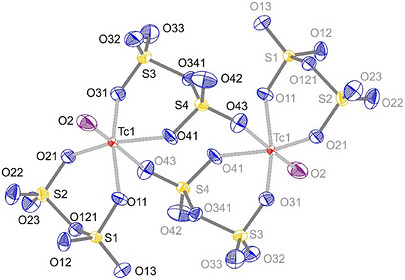
The dimeric anion in the crystal structure of (NH_4_)_2_[Tc_2_O_2_(S_2_O_7_)_4_]. The anion shows C_i_ symmetry and the atoms generated by the inversion centre are labelled in light grey colour. The ellipsoids are shown at 90% probability. Selected bond lengths (pm) and angles (°); theoretical values given in *italics*: Tc1‒O2 161.0(3)/*164.2*, Tc1‒O11 200.4(3)/*200.6*, Tc1‒O21 199.8(3)/*198.2*, Tc1‒O31 200.6(3)/*203.8*, Tc1‒O41 201.6(3)/*204.4*, Tc1‒O43 225.0(3)/*229.0*; O2‒Tc1‒O43 176.4(2)/*178.4* (full data can be found in the Supporting Information).

During the reaction of the colorless pertechnetate with SO_3_, an orange solid forms upon contact with SO_3_, which turns dark blue after a while with formation of single crystals (see Supporting Information for details). According to our findings for the reaction of ammonium pertechnetate with triflic acid we assume that in a first step a Tc(VII) sulfate forms, which is then reduced to the Tc(V) sulfate (NH_4_)_2_[Tc_2_O_2_(S_2_O_7_)_4_], probably under formation of oxygen, as previously reported [17]. The structure determination of the latter revealed the formation of the dimeric anion [Tc_2_O_2_(S_2_O_7_)_4_]^2−^, exhibiting inversion symmetry (Figure 1). In the anion, each Tc atom is coordinated by oxygen atoms of three disulfate anions and one oxide ligand. The (S_2_O_7_)^2−^ anions belong to two crystallographically different species, represented by the sulfur atoms S1/S2 and S3/S4, respectively. One disulfate group (S1/S2) acts as a terminal chelating ligand, the second anion (S3/S4) is chelating to one technetium atom and monodentate to the second one in the dimeric unit. The distances Tc─O to the oxygen atoms of the chelating (S_2_O_7_)^2−^ groups (O11, O21, O31, O41) are found uniformly around 200 pm while the oxygen atom of the monodentate anion (O43) is found at a longer distance of 225.0(3) pm. The oxide ligand (O2) is bonded to the Tc atom with a short distance of 161.0(3) pm. It is placed opposite to O43, leading to the increased bond length Tc‐O43 according to the *trans* effect [19]. The angle O2‒Tc1‒O43 shows a value of 176.4(2)°, that is the moiety is almost linear. Within the (S_2_O_7_)^2−^ ions the bond lengths S─O vary with respect to the connectivity of the oxygen atoms. Those atoms which are closely bonded to the Tc atoms show distances S─O of about 150 pm while the nonbonded ones reveal values around 141 pm. For the oxygen atom with the longest bond to the Tc atom (O43) a distance S─O of 143.7(3) pm is found. The observed distances Tc─O are well in line with the observations in (NH_4_)_2_[TcO(CF_3_SO_3_)_5_](CF_3_SO_3_H) [17]. In the latter, the oxide ligand shows a distance of 161.2(2) pm and the monodentate triflate anions coordinated to the Tc atom with distances between 201.0(2) and 202.5(2) pm.

The structure of the dimeric anion and the observed bond lengths and angles are well reproduced by quantum mechanical calculations within DFT theory (see Figure 1 and full data in the Supporting Information). The calculated values are slightly larger by about 2% compared to the experimental data for most of the distances. In the crystal structure of (NH_4_)_2_[Tc_2_O_2_(S_2_O_7_)_4_] the inversion center of the anion is located on the *Wyckoff* site 2*d* with the coordinates ½ 0 ½ and ½ ½ 0, respectively (see Figure S3 in the supporting information). Charge balance for the dimeric anions is achieved by two NH_4_^+^ cations which are crystallographically identical. The (NH_4_)^+^ cations are surrounded by eight oxygen atoms in a range between 281 and 321 pm for the N‐O distances. The coordination polyhedron is a slightly distorted dodecahedron.

The normal modes observed in the Raman spectrum of (NH_4_)_2_[Tc_2_O_2_(S_2_O_7_)_4_] can be assigned based on the data from the DFT calculation. Accordingly, the stretching and deformation modes for the [S_2_O_7_]^2−^ anions are found at 1232 and 1366 cm^−1^, respectively (*ν*_asym._ S–O), 1155 cm^−1^ (*ν*_sym._ S–O), and between 506 and 660 cm^−1^ (*δ* SO_3_) (cf. Supporting Information, Table S19). The stretching modes Tc─O to the terminal oxygen ligands are found at 1053, 1026, and 936 cm^−1^, while the respective values to the oxygen atoms of the disulfate groups occur around 750 cm^−1^. These values are in line with the observation for other sulfates and polysulfates [20, 21, 22], and especially the Tc─O vibration matches the findings for mono‐oxido technetium complexes, for example {(μ‐O)[TcX(bpy)_2_]_2_}^2+^ (X = Cl, Br, bpy = bipyridine) [23].

**FIGURE 2:**
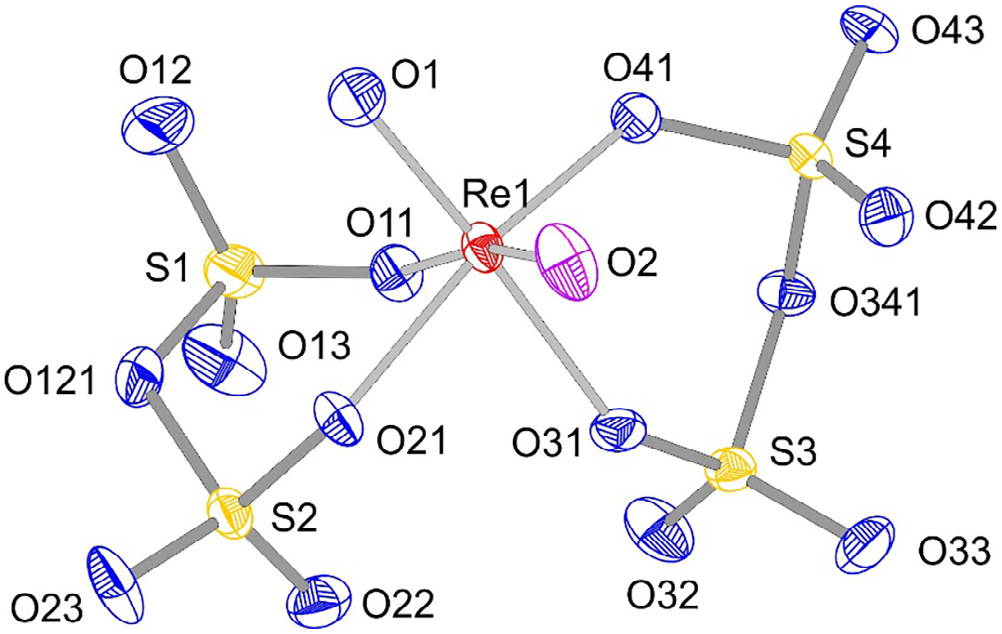
One of the crystallographically independent [ReO_2_(S_2_O_7_)_2_]^−^ anions in the crystal structure of (NH_4_)[ReO_2_(S_2_O_7_)_2_]. The ellipsoids are shown at 90% probability. Selected bond distances (pm) and angles (°); theoretical values given in *italics*: Re1‒O1 167.8(6)/*168.2*, Re1‒O2 168.2(7)/*168.1*, Re1‒O11 215.0(6)/*214.7*, Re1‒O21 194.6(6)/*194.1*, Re1‒O31 208.2(5)/*207.8*, Re1‒O41/ 193.3(6)/*193.1*; O1‒Re1‒O31 166.0(3)/165.9, O2‒Re1‒O11 168.6(3)/*168.6*.

In contrast to the reaction of SO_3_ with (NH_4_)(TcO_4_), the respective reaction of the perrhenate (NH_4_)(ReO_4_) leads at 80 °C to yellow crystals of (NH_4_)[ReO_2_(S_2_O_7_)_2_], according to (NH_4_)(ReO_4_) +4 SO_3_ → (NH_4_)[ReO_2_(S_2_O_7_)_2_]. Even prolonged reaction times gave this Re(VII) product and in no case any reduction was observed. Also, in aqueous solution, the +VII oxidation state is most prominent for rhenium, even if a recent report shows that reduction under formation of a Re(VI) species occurs upon evaporation of perrhenic acid [24]. In the orthorhombic crystal structure (space group *Pca*2_1_) of (NH_4_)[ReO_2_(S_2_O_7_)_2_], two crystallographically different [ReO_2_(S_2_O_7_)_2_]^−^ anions with *C*_1_ symmetry can be found. Both show the rhenium atoms in octahedral coordination of two chelating disulfate groups and two oxygen atoms (Figure 2). Details shall be discussed for one of the almost identical complexes, full data for both species can be found in the Supporting Information. The oxide ligands in the [ReO_2_(S_2_O_7_)_2_]^−^ anion are in *cis* orientation with respect to each other and show distances Re─O of 167.8(6) and 168.2(7) pm, respectively. The chelating [S_2_O_7_]^2−^ anions are bonded via oxygen atoms to the central rhenium atom. The observed Re─O bonds are significantly longer for those oxygen atoms that are oriented *trans* to the two oxide ligands. This *trans* effect is well‐known in the chemistry of high‐valent transition metals [19]. The Re─O bonds in *trans*‐position show values of 215.0(6) and 208.2(5) pm while the remaining bond lengths are found at 194.6(6) and 193.3(6) pm. An analogous behavior has been observed for sulfates of heptavalent rhenium [20, 21, 22]. The distances are well reproduced by theoretical calculations (cf. supporting information). Charge compensation for the [ReO_2_(S_2_O_7_)_2_]^−^ complexes is achieved by two crystallographically different (NH_4_)^+^ cations, which are surrounded by eight and nine oxygen atoms, respectively, if distances up to 327 pm are considered.

The fundamental vibrations of the [ReO_2_(S_2_O_7_)_2_]^−^ complex as observed in the Raman spectrum can be assigned based on the data from the DFT calculation. Accordingly, the stretching and deformation modes for the [S_2_O_7_]^2−^ groups are found between 1224 and 1402 cm^−1^ (*ν*_asym._ S–O and *ν*_sym._ S–O), and between 556 and 620 cm^−1^ (*δ* SO_3_) (cf. Supporting information, Table S20). The stretching modes Re─O to the terminal oxygen ligands are found at 1066, 1018, and 962 cm^−1^, while the respective values to the oxygen atoms of the disulfate groups are seen around 790 cm^−1^.

## 3 Conclusion

We have shown that even in the reaction with the strong oxidizer SO_3_ the pertechnetate anion (TcO_4_)^−^ is reduced to Tc(V) under formation of (NH_4_)_2_[Tc_2_O_2_(S_2_O_7_)_4_]. The unique structure of the compound exhibits the dimeric complex [Tc_2_O_2_(S_2_O_7_)_4_]^2−^ with tridentate bridging disulfate groups. Interestingly, the perrhenate anion (ReO_4_)^−^ does not undergo reduction under similar reaction conditions but gave the Re(VII) complex [ReO_2_(S_2_O_7_)_2_]^−^ instead. On one hand, our findings show that the group 7 metals technetium and rhenium are by far not as similar as often believed. Thus, scrutiny of technetium chemistry is still highly needed. On the other hand, the behavior of technetium in concentrated Brønsted acids and their anhydrides shows that Tc(V) compounds are the dominating species in these systems. The structural identification of these species is of great values with respect to the separation of the metal during nuclear waste treatment, which includes treatment of the spent fuel with mineralic acids.

## 4 Experimental Section

In a controlled area suitable for handling the ^99^Tc isotope, (NH_4_)(TcO_4_) was reacted with SO_3_ and the pyridine adduct py·SO_3_ in a Schlenk tube. As a product of the reaction at 80°C blue single crystals of the Tc(V) sulfate (NH_4_)_2_[Tc_2_O_2_(S_2_O_7_)_4_] were obtained. For comparision, a similar reaction was conducted with (NH_4_)(ReO_4_). In this case, yellowish single crystals of the Re(VII) sulfate (NH_4_)[ReO_2_(S_2_O_7_)_2_] are formed. Single crystal structure determinations of both compounds have been performed on a Bruker D8 VENTURE KAPPA diffractometer using MoK_α_ radiation (71.073 pm). The intensity data were processed using the programs APEX4, SAINT and SADABS [25, 26, 27]. The structure solution was performed using the SHELXL system [28]. Selected crystallographic data are summarized in Table 1. Furthermore, supplementary crystallographic data for this paper can be obtained free of charge from the joint Cambridge Crystallographic Data Centre and Fachinformationszentrum Karlsruhe Access Structures service on quoting the deposition numbers 2409908 ((NH_4_)_2_[Tc_2_O_2_(S_2_O_7_)_4_]), and 2454854 ((NH_4_)[ReO_2_(S_2_O_7_)_2_]) [29]. Raman spectra were recorded with an inVia Qotor confocal RAMAN microscope by Renishaw GmbH (Pliezhausen, Germany), equipped with 10x, 50x, and 100x magnification lenses. The spectra were measured on selected single crystals with a green laser (532 nm, 100 mW) with an exposure time of 10 s. The data was processed and corrected with the WiRE 5.1 software. Quantum mechanical calculations were performed using the ORCA program [30]. Full details of synthesis, characterization and quantum chemical calculations can be found in the Supporting Information.

**TABLE 1:** Crystallographic data of (NH_4_)_2_[Tc_2_O_2_(S_2_O_7_)_4_] and (NH_4_)[ReO_2_(S_2_O_7_)_2_].^a^

Compound	(NH_4_)_2_[Tc_2_O_2_(S_2_O_7_)_4_]	(NH_4_)[ReO_2_(S_2_O_7_)_2_]
T/K	100.0(2)	100.0(2)
Crystal System	*monoclinic*	*orthorhombic*
Space group	*P*2_1_/*c*	*Pca*2_1_
*a/*pm	775.67(5)	1842.7(1)
*b*/pm	1339.55(8)	847.03(5)
*c/*pm	1233.32(8)	1663.8(1)
*β/*°	100.566(2)	
*V*/nm^3^	12.591(1)	25.969(3)
Z	2	8
Final *R* indexes [all data]	*R*_1_ = 0.0591, *wR*_2_ = 0.1157	*R*_1_ = 0.0513, *wR*_2_ = 0.0871
*R*_int_	0.0638	0.0512
Goof	1.268	1.030
CCDC number	2409908	2454854
Twin law		−1 0 0, 0 ‐1 0, 0 0 ‐1
BASF		0.313(8)
Flack x		0.313(8)

^a^ Full data can be found in the Supporting Information.

## Conflicts of Interest

The authors declare no conflict of interest.

## Supporting information




**Supporting file 1**: chem70498‐sup‐0001‐SuppMat.pdf.

## Data Availability

The data that support the findings of this study are available from the corresponding author upon reasonable request.
