# Supplementary material for: (NH4)2[Tc2O2(S2O7)4]: An Unexpected Technetium(V)‐Sulfate From the Reaction of (NH4)(TcO4) With SO3
Source: Chemistry. 2025 Nov 30;32(29):e02493. doi: 10.1002/chem.202502493 (PMC13451577; doi:10.1002/chem.202502493)
Supplement: Supplementary file 1 [file CHEM-32-e02493-s001.pdf]

Supporting Information

$(NH_4)_2[Tc_2O_2(S_2O_7)_4]$: An unexpected Technetium(V)-sulfate from the reaction of $(NH_4)[TcO_4]$ with SO_3

Stefan Sutorius, Julian Heinen, David van Gerven, Jörn Bruns, Erik Strub, Mathias S. Wickleder

Table of Contents

A. Syntheses
B. Structure determination and crystallographic details
C. Powder XRD
D. Vibrational Spectroscopy
E. Results from quantum chemical calculations

A. Syntheses

(1) $(NH_4)_2[Tc_2O_2(S_2O_7)_4]$: 20 µL of a $(NH_4)(TcO_4)$ solution (1.87 mg/20 µL, 10.4 µmol) was placed as a drop on the wall of a Schlenk tube and dried in a drying oven at 90 °C, leaving colourless $(NH_4)(TcO_4)$ on the glass wall. 15 mg Py·SO_3 (61 µmol, *SIGMA-ALDRICH Co.*, St. Louis, USA) was placed at the bottom of the glass tube and SO_3 was added. After adding SO_3, the pertechnetate at the glass wall of the tube turned orange. The orange reaction product turned blue during the reaction on a sand bath (80 °C for 20 h). The blue reaction product consists of a number of single crystals, suitable for X-ray structure elucidation (Figure S1).

(2) $(NH_4)[ReO_2(S_2O_7)_2]$: $(NH_4)(ReO_4)$ (200 mg, 0,738 mmol; 1,00 eq., BASF) was placed in a glass ampoule and 0.42 mL (10 mmol; 13,6 eq.) of freshly distilled SO_3 was added. The ampoule was torch sealed and heated to 130 °C within 10 h in a tube furnace. This temperature was maintained for 24 h and the reaction mixture was then allowed to cool down to room temperature within 48 h. The compound could be obtained in form of yellow highly moisture sensitive crystals, which had to be handled under inert gas conditions. One of these crystals was analysed by X-ray structure determination.

SO_3 generation: SO_3 was produced using a specially designed setup for generation, distillation, and subsequent transfer into glass ampoules under a nitrogen atmosphere. For this, fuming sulfuric acid (10 mL, 65% SO_3, used as received; VWR Chemicals, Darmstadt, Germany) was slowly added via a dropping funnel into a 250 mL flask containing P_4O_{10} (200 g, >99%; Carl Roth, Karlsruhe, Germany). Simultaneously, the flask was heated to 135 °C, causing the generated SO_3 to distil into a connected burette (graduated to 0.01 mL ±0.01). A glass ampoule (200 mm length, 16 mm diameter, 1.80 mm wall thickness) was used for the synthesis of **$(NH_4)[ReO_2(S_2O_7)_2]$**. For the preparation of **$(NH_4)_2[Tc_2O_2(S_2O_7)_4]$** SO_3 was filled into a Schlenk tube (Figure S2) which was transferred to the control area. Here the SO_3 was sublimed into the reaction vessel containing $(NH_4)(TcO_4)$.

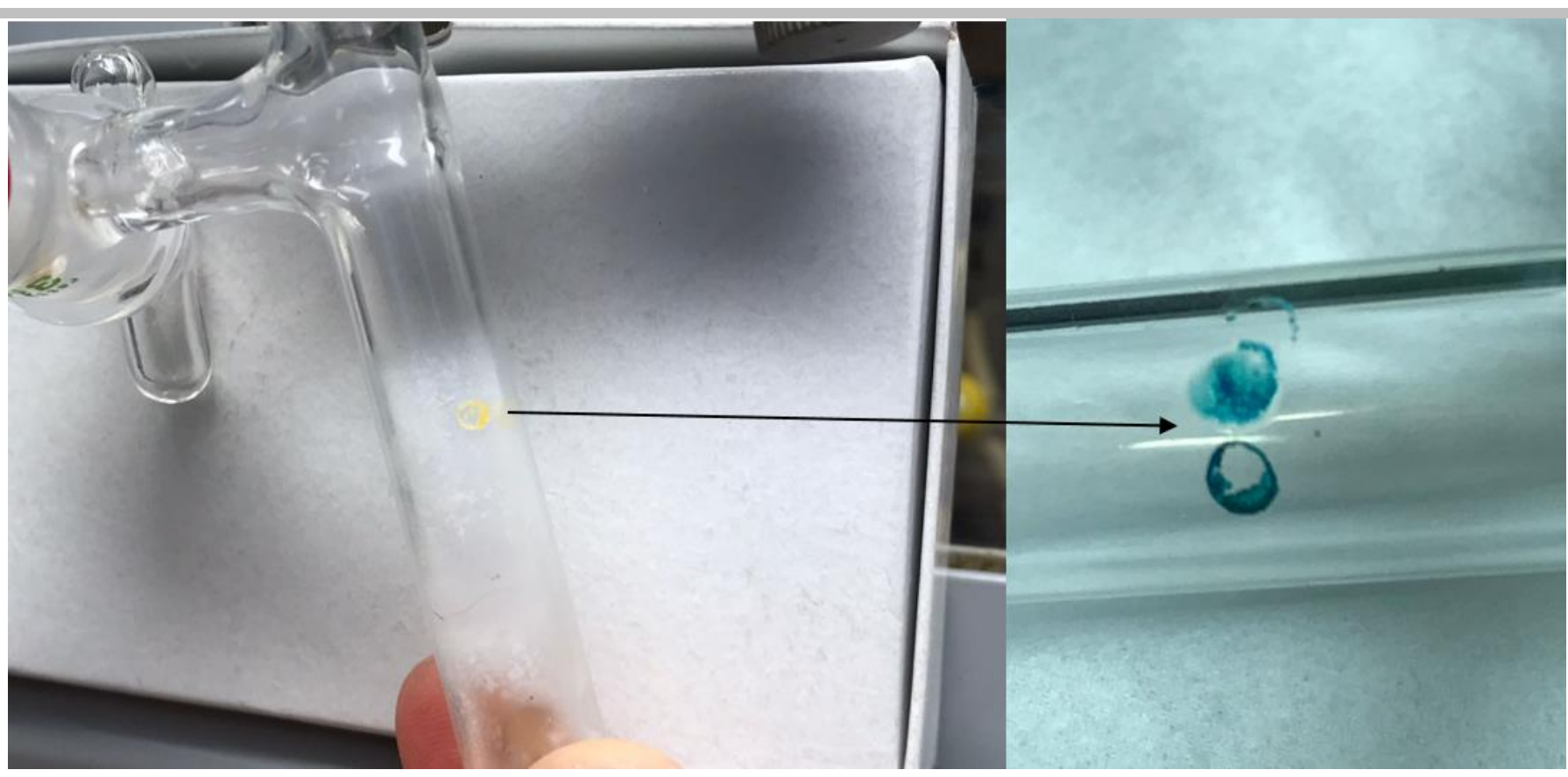

Figure S1: $(NH_4)(TcO_4)$ turning orange after adding SO_3 (left). After heating, it turned blue (right).

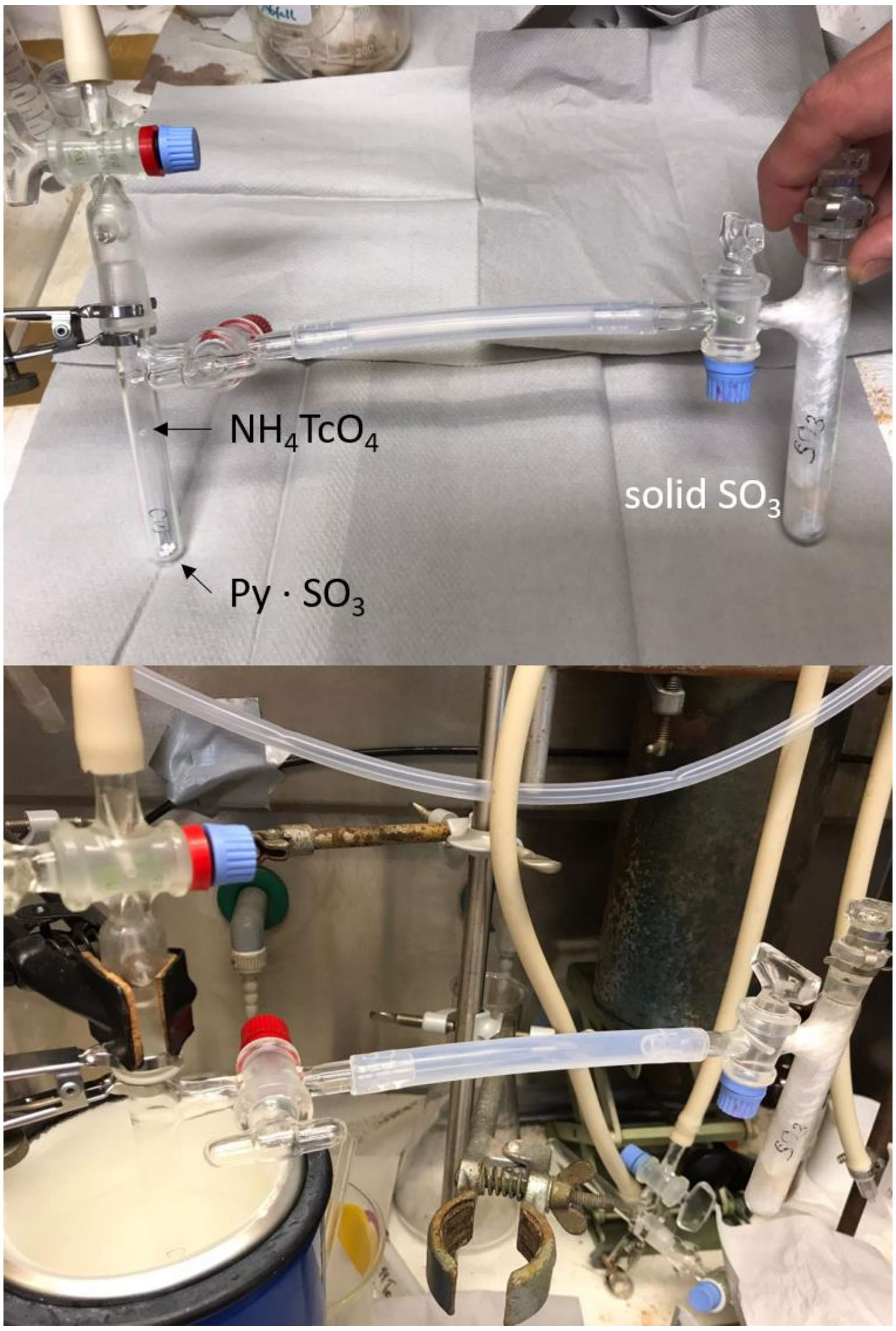

Figure S2: In the top part, the setup is shown. A Schlenk-tube with solid SO_3 is connected to a Schlenk-tube with $Py{\cdot}SO_3$ and $(NH_4)(TcO_4)$ via an FEP bridge. By heating the Schlenk tube with SO_3, it is sublimed and then re-sublimed to the Schlenk-tube containing $(NH_4)(TcO_4)$.

B. Structure determination and crystallographic details

Single-crystal structure analysis was conducted using a Bruker D8 VENTURE KAPPA diffractometer equipped with a microfocus sealed tube, a multilayer mirror monochromator, and a Bruker PHOTON III detector. MoKα radiation (71.073 pm) served as the X-ray source. Crystals were prepared in perfluorinated ether (Fomblin® YR-1800) and selected under a polarized light microscope. They were mounted on a micromount with a 150 µm polymer loop and aligned with the X-ray beam at a controlled temperature of 100 K. Intensity data collection and image processing were performed using APEX4 software.[1] Data integration was carried out with SAINT, and a multi-scan absorption correction was applied using SADABS.[2,3] The structure was solved by intrinsic phasing (SHELXT) within the Olex2 software environment and refined using least-squares methods in SHELXL.[4]

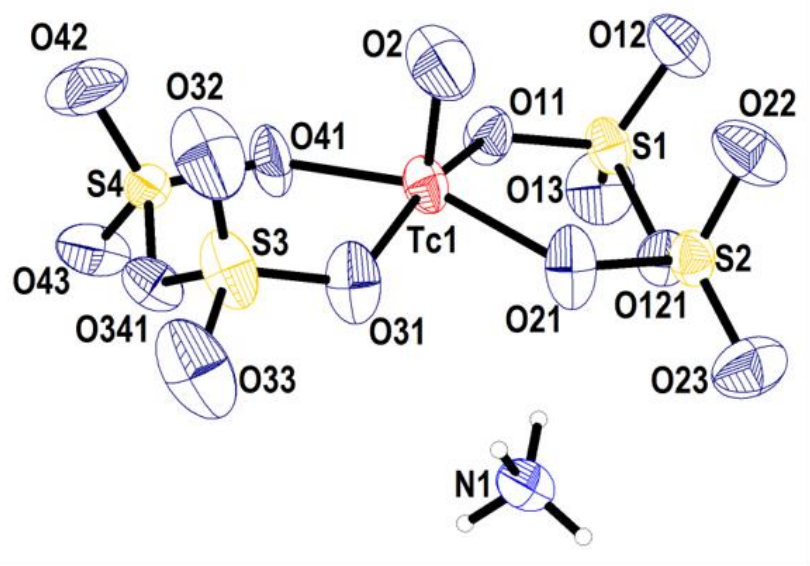

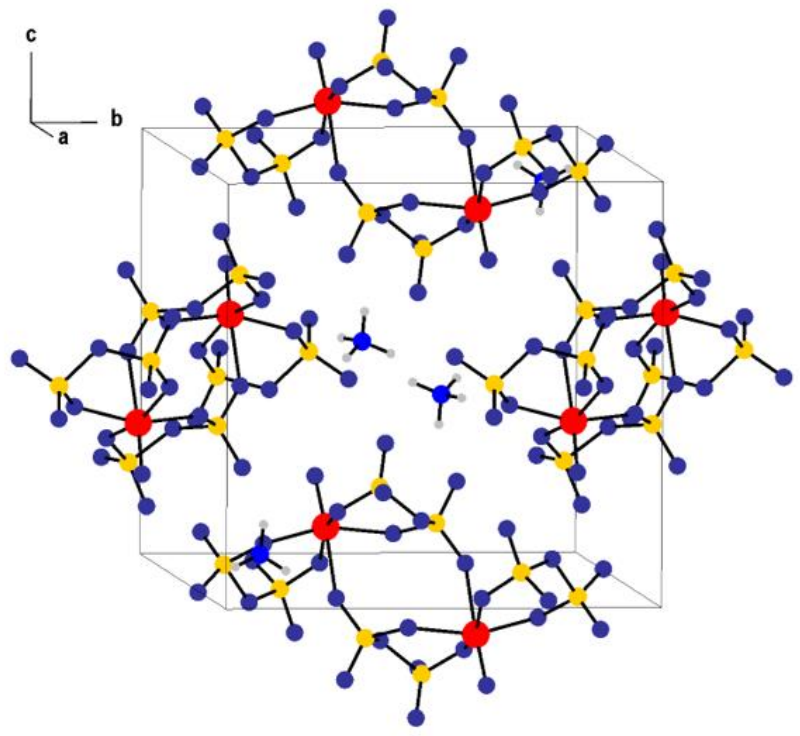

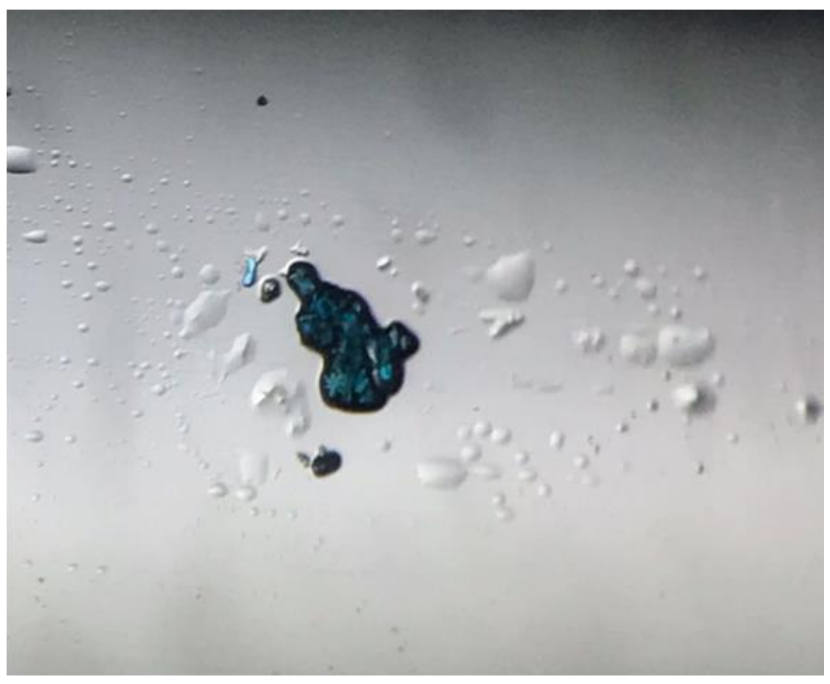

Figure S3: Structure and labelling with ellipsoids shown at 90% probability (left), crystal structure of cell (middle) and microscope image of crystals (right) of **$(NH_4)_2[Tc_2O_2(S_2O_7)_4]$**.

Table S1: Crystallographic and refinement data for **$(NH_4)_2[Tc_2O_2(S_2O_7)_4]$**.

Empirical formula	$H_8N_2O_{30}S_8Tc_2$
Formula weight	968.56
Temperature/K	100
Crystal system	monoclinic
Space group	$P2_1/c$
a/Å	7.7567(5)
b/Å	13.3955(8)
c/Å	12.3332(8)
β/°	100.566(2)
Volume/Å^3	1259.8(1)
Z	2
ρ_{calc}g/cm^3	2.553
μ/mm^{-1}	1.892
F(000)	952
Crystal size/mm^3	0.207 × 0.152 × 0.144
Radiation	MoKα (λ = 0.71073)
2Θ range for data collection/°	5.342 to 66.274
Index ranges	$-11 \leq h \leq 11$, $-20 \leq k \leq 20$, $-18 \leq l \leq 18$
Reflections collected	145777

Independent reflections	4778 [R_{int} = 0.0638, R_{sigma} = 0.0193]
Data/restraints/parameters	4778/10/206
Goodness-of-fit on F^2	1.269
Final R indexes [I>=2σ (I)]	R_1 = 0.0531, wR_2 = 0.1126
Final R indexes [all data]	R_1 = 0.0588, wR_2 = 0.1147
Largest diff. peak/hole / e Å^{-3}	1.33/-1.65
CCDC/ICSD no.	2409908

Table S2: Atomic coordinates and equivalent isotropic displacement parameters (Å^2 x 10^3) for **$(NH_4)_2[Tc_2O_2(S_2O_7)_4]$**. U(eq) is defined as one third of the trace of the orthogonalized U_{ij} tensor**.**

**Atom**	***x***	***y***	***z***	**U(eq)**
S1	-92(1)	1765.0(7)	4150.2(8)	12.4(2)
S2	1971(1)	3468.1(7)	4996.8(8)	13.8(2)
S3	6954(1)	879.6(9)	7455.0(9)	20.0(2)
S4	5089(1)	-787.8(7)	6358.7(7)	12.0(2)
O2	2409(5)	1468(3)	7131(3)	28.9(8)
O11	1128(4)	1097(2)	4919(2)	15.8(5)
O12	-1513(4)	2107(2)	4630(3)	20.1(6)
O13	-450(4)	1351(3)	3074(3)	21.6(6)
O21	3393(4)	2837(2)	5669(3)	21.1(6)
O22	650(4)	3734(3)	5595(3)	24.2(7)
O23	2796(5)	4219(3)	4456(3)	24.7(7)
O31	5801(4)	1618(2)	6728(3)	21.6(6)
O32	6218(5)	607(3)	8389(3)	32.7(9)
O33	8733(5)	1152(3)	7557(4)	34.3(9)
O41	3628(4)	-94(2)	5928(3)	19.3(6)
O42	4819(5)	-1310(3)	7304(3)	28.9(8)
O43	5480(4)	-1387(3)	5468(3)	20.7(6)
O121	1162(4)	2719(2)	4003(2)	14.9(5)
O341	6806(4)	-110(2)	6642(2)	15.1(5)
Tc1	3279.1(4)	1391.0(2)	6044.5(3)	13.27(8)
N1	7146(5)	3662(3)	5909(3)	17.7(6)
H1A	7754.98	3190.01	5602.38	21
H1B	7375.48	4274.81	5649.28	21
H1C	7482.08	3649.41	6654.59	21
H1D	6033.48	3416.11	5849.08	21

Table S3: Anisotropic displacement parameters (Å^2 × 10^3) for **$(NH_4)_2[Tc_2O_2(S_2O_7)_4]$**. The anisotropic displacement factor exponent takes the form: $-2\pi^2$ [$h^2 a^{*2} U_{11}$ + 2 h k a* b* U_{12} +…].

**Atom**	**U_{11}**	**U_{22}**	**U_{33}**	**U_{23}**	**U_{13}**	**U_{12}**
S1	9.9(3)	13.0(4)	13.0(4)	-1.9(3)	-1.4(3)	1.9(3)
S2	13.1(4)	10.1(4)	17.8(4)	-0.8(3)	1.7(3)	0.6(3)
S3	16.6(4)	23.0(5)	16.9(4)	-7.6(4)	-6.3(3)	5.4(4)
S4	11.1(3)	13.8(4)	11.3(3)	2.5(3)	2.6(3)	1.3(3)
O2	30(2)	43(2)	15(1)	5(1)	6(1)	15(2)

O11	12(1)	15(1)	19(1)	4(1)	-2(1)	0(1)
O12	13(1)	18(1)	30(2)	-8(1)	5(1)	0(1)
O13	24(2)	23(2)	16(1)	-5(1)	-1(1)	-1(1)
O21	21(1)	14(1)	24(2)	-4(1)	-8(1)	2(1)
O22	19(1)	25(2)	31(2)	-12(1)	9(1)	-1(1)
O23	28(2)	21(2)	26(2)	0(1)	8(1)	-8(1)
O31	19(1)	16(1)	25(2)	-5(1)	-7(1)	2(1)
O32	38(2)	45(2)	13(1)	-5(1)	1(1)	17(2)
O33	15(1)	35(2)	47(2)	-18(2)	-10(1)	0(1)
O41	11(1)	15(1)	30(2)	1(1)	-3(1)	5(1)
O42	25(2)	42(2)	20(2)	15(2)	4(1)	-4(2)
O43	18(1)	23(2)	23(1)	-6(1)	9(1)	-5(1)
O121	15(1)	13(1)	16(1)	-1(1)	1(1)	-2(1)
O341	11(1)	18(1)	16(1)	-6(1)	1.5(9)	-2(1)
Tc1	12.7(1)	14.6(1)	11.7(1)	-1.0(1)	-0.16(9)	3.7(1)
N1	15(1)	20(2)	19(2)	0(1)	6(1)	2(1)

Table S4: Experimental bond lengths [Å] for **$(NH_4)_2[Tc_2O_2(S_2O_7)_4]$**.

**Atom–Atom**	**Length [Å]**
S1–O11	1.506(3)
S1–O12	1.419(3)
S1–O13	1.418(3)
S1–O121	1.636(3)
S2–O21	1.511(3)
S2–O22	1.413(3)
S2–O23	1.423(3)
S2–O121	1.619(3)
S3–O31	1.512(3)
S3–O32	1.424(4)
S3–O33	1.410(4)
S3–O341	1.654(3)
S4–O41	1.486(3)
S4–O42	1.408(3)
S4–O43	1.438(3)
S4–O341	1.597(3)
O2–Tc1	1.610(3)
O11–Tc1	2.004(3)
O21–Tc1	1.998(3)
O31–Tc1	2.006(3)
O41–Tc1	2.016(3)
O43–Tc1$^{\#1}$	2.250(3)
N1–H1A	0.88(1)
N1–H1B	0.88(1)
N1–H1C	0.88(1)
N1–H1D	0.88(1)

#1: 1-*x*,-*y*,1-*z*

Table S5: Experimental bond angles [°] for **$(NH_4)_2[Tc_2O_2(S_2O_7)_4]$**.

**Atom–Atom–Atom**	**Angle [°]**
O11–S1–O121	102.4(2)
O12–S1–O11	112.1(2)
O12–S1–O121	108.5(2)
O13–S1–O11	110.5(2)
O13–S1–O12	118.9(2)
O13–S1–O121	102.7(2)
O21–S2–O121	102.2(2)
O22–S2–O21	112.4(2)
O22–S2–O23	119.9(2)
O22–S2–O121	109.5(2)
O23–S2–O21	107.9(2)
O23–S2–O121	103.2(2)
O31–S3–O341	101.3(2)
O32–S3–O31	111.5(2)
O32–S3–O341	106.9(2)
O33–S3–O31	110.3(2)
O33–S3–O32	121.6(3)
O33–S3–O341	102.7(2)
O41–S4–O341	105.9(2)
O42–S4–O41	112.8(2)
O42–S4–O43	116.1(2)
O42–S4–O341	110.3(2)
O43–S4–O41	109.2(2)
O43–S4–O341	101.6(2)
S1–O11–Tc1	131.9(2)
S2–O21–Tc1	127.6(2)
S3–O31–Tc1	125.1(2)
S4–O41–Tc1	134.0(2)
S4–O43–Tc1	144.8(2)
S2–O121–S1	123.8(2)
S4–O341–S3	123.4(2)
O2–Tc1–O11	99.6(2)
O2–Tc1–O21	100.0(2)
O2–Tc1–O31	99.6(2)
O2–Tc1–O41	101.8(2)
O2–Tc1–O43	176.4(2)
O11–Tc1–O31	160.8(1)
O11–Tc1–O41	82.1(1)
O11–Tc1–O43	81.6(1)
O21–Tc1–O11	95.5(1)
O21–Tc1–O31	82.5(1)
O21–Tc1–O41	158.1(1)

O21–Tc1–O43	76.5(1)
O31–Tc1–O41	92.6(1)
O31–Tc1–O43	79.4(1)
O41–Tc1–O43	81.7(1)
H1A–N1–H1B	103(6)
H1A–N1–H1C	117(7)
H1A–N1–H1D	100(7)
H1B–N1–H1C	99(7)
H1B–N1–H1D	120(7)
H1C–N1–H1D	118(8)

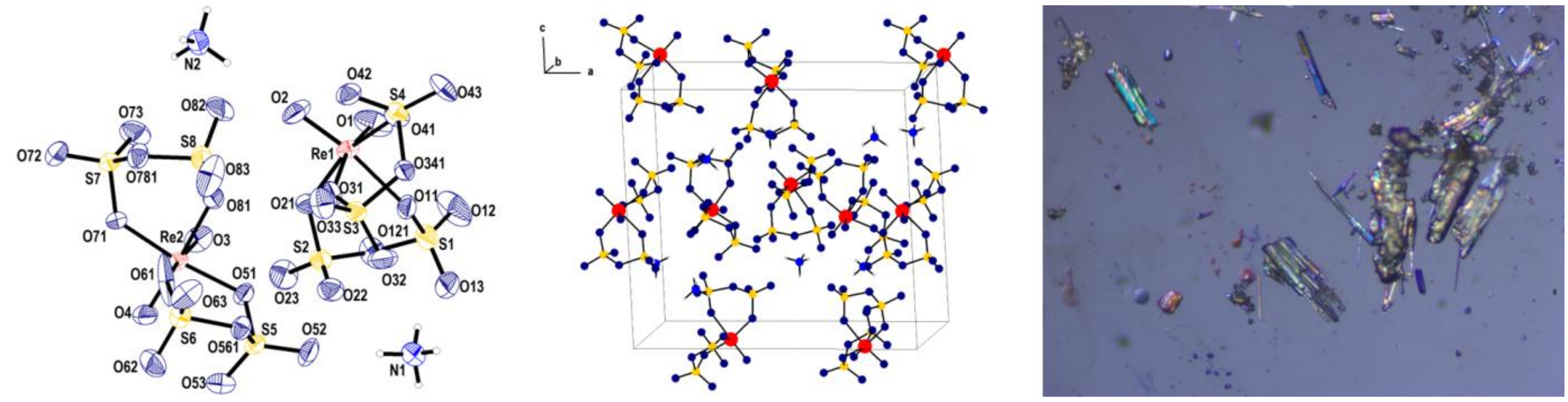

Figure S4: Structure and labelling with ellipsoids shown at 90% probability(left), crystal structure of cell (middle) and microscope image of crystals (right) of **$(NH_4)[ReO_2(S_2O_7)_2]$**.

Table S6: Crystallographic and refinement data for **$(NH_4)[ReO_2(S_2O_7)_2]$**

Identification code	NH4[ReO2(S2O7)2]
Empirical formula	$H_4NO_{16}ReS_4$
Formula weight	588.48
Temperature/K	100.0
Crystal system	orthorhombic
Space group	$Pca2_1$
a/Å	18.427(1)
b/Å	8.4703(5)
c/Å	16.638(1)
α/°	90
β/°	90
γ/°	90
Volume/Å^3	2596.9(3)
Z	8
ρ_{calc}g/cm^3	3.010
μ/mm^{-1}	10.095
F(000)	2224
Crystal size/mm^3	0.196 × 0.179 × 0.141
Radiation	MoKα (λ = 0.71073)

2Θ range for data collection/°	4.422 to 55.994
Index ranges	$-24 \leq h \leq 24$, $-11 \leq k \leq 11$, $-21 \leq l \leq 21$
Reflections collected	69093
Independent reflections	6208 [R_{int} = 0.0393, R_{sigma} = 0.0209]
Data/restraints/parameters	6208/1/380
Goodness-of-fit on F^2	1.146
Final R indexes [I>=2σ (I)]	R_1 = 0.0211, wR_2 = 0.0516
Final R indexes [all data]	R_1 = 0.0212, wR_2 = 0.0516
Largest diff. peak/hole / e Å^{-3}	2.94/-1.02
Twin law	-1 0 0, 0 -1 0, 0 0 -1
Flack x	0.313(8)
CCDC/ICSD no.	2454854

Table S7: Atomic coordinates and equivalent isotropic displacement parameters (Å^2 x 10^3) for **$(NH_4)[ReO_2(S_2O_7)_2]$**. U(eq) is defined as one third of the trace of the orthogonalized U_{ij} tensor**.**

**Atom**	***x***	***y***	***z***	U_{eq}
Re1	4692.2(2)	9795.1(3)	5308.4(2)	9.69(7)
S1	5524(1)	9890(3)	3542(1)	18.0(4)
S2	4024(1)	9242(2)	3557(1)	12.3(4)
S3	4973.0(9)	5856(2)	5313(1)	11.7(3)
S4	5688(1)	7761(2)	6456(1)	11.9(3)
O1	4960(4)	11672(7)	5181(4)	23(1)
O2	4072(4)	9933(8)	6047(4)	19(1)
O11	5406(3)	9148(8)	4340(3)	15(1)
O12	5867(4)	11381(9)	3557(5)	33(2)
O13	5768(4)	8748(10)	2971(4)	28(2)
O21	3970(3)	9689(7)	4457(3)	12(1)
O22	4215(3)	7639(8)	3460(4)	19(1)
O23	3394(4)	9876(9)	3182(4)	24(2)
O31	4555(3)	7357(6)	5260(4)	14(1)
O32	5242(3)	5297(8)	4568(4)	21(1)
O33	4621(3)	4791(7)	5835(4)	19(1)
O41	5536(3)	9270(7)	5946(4)	13(1)
O42	5085(3)	7461(7)	6957(3)	15(1)
O43	6402(3)	7868(7)	6754(4)	19(1)
O121	4673(4)	10337(8)	3265(4)	20(1)
O341	5727(3)	6452(7)	5763(3)	12(1)
Re2	2276.7(2)	6226.4(3)	4704.8(2)	9.32(7)
S5	3305(1)	3972(2)	3699(1)	11.1(3)
S6	2490(1)	2279(2)	4856(1)	12.7(4)
S7	1532(1)	7107(2)	6371(1)	12.6(4)
S8	2953(1)	5908(3)	6580(1)	16.4(4)
O3	2461(3)	8144(7)	4506(4)	18(1)
O4	1696(3)	5712(8)	3967(3)	15(1)
O51	3165(3)	5503(7)	4185(3)	12(1)
O52	4037(3)	3992(7)	3446(4)	19(1)

O53	2721(3)	3718(7)	3155(4)	17(1)
O61	2261(3)	3876(7)	5082(4)	16.9(6)
O62	2023(3)	1628(7)	4273(4)	16.9(6)
O63	2731(3)	1330(6)	5508(4)	16.9(6)
O71	1509(3)	6492(7)	5490(3)	12(1)
O72	843(3)	6814(8)	6719(4)	21(1)
O73	1848(4)	8626(7)	6390(4)	22(1)
O81	2953(3)	6402(7)	5716(3)	14(1)
O82	3252(4)	7123(10)	7069(4)	32(2)
O83	3160(4)	4329(9)	6701(4)	24(2)
O561	3275(3)	2682(7)	4395(4)	16.9(6)
O781	2068(3)	5866(8)	6775(3)	16(1)
N1	5386(4)	5320(9)	2796(5)	20(2)
N2	2809(4)	10911(9)	7219(4)	17(1)
H1A	5434.35	6341.36	2966.87	25
H1B	5769.35	4745.66	2966.87	25
H1C	5369.05	5312.76	2247.27	25
H1D	4957.85	4903.86	2997.97	25
H2A	2685.16	10085.23	7531.93	20
H2B	2414.66	11228.83	6924.43	20
H2C	2957.76	11736.14	7531.93	20
H2D	3180.66	10629.83	6870.33	20

Table S8: Anisotropic displacement parameters ($Å^2 \times 10^3$) for **$(NH_4)[ReO_2(S_2O_7)_2]$**. The anisotropic displacement factor exponent takes the form: $-2\pi^2$ [$h^2 a^{*2} U_{11} + 2 h k a^* b^* U_{12} + \ldots$].

**Atom**	U_{11}	U_{22}	U_{33}	U_{23}	U_{13}	U_{12}
Re1	12.5(1)	9.3(1)	7.3(1)	-0.1(1)	-1.1(1)	3.43(9)
S1	14.4(9)	27(1)	13(1)	2.6(8)	1.2(7)	-9.2(8)
S2	12.6(8)	16.1(9)	8.3(8)	1.2(7)	-1.0(6)	-1.0(7)
S3	12.1(7)	9.7(7)	13.4(8)	-2.1(8)	-1.6(8)	-0.5(6)
S4	12.9(8)	12.5(8)	10.4(8)	-0.1(7)	-2.9(6)	-0.1(7)
O1	38(4)	5(2)	25(4)	1(2)	-12(3)	-1(2)
O2	21(3)	26(3)	9(3)	-3(2)	3(2)	8(3)
O11	7(2)	25(3)	11(3)	4(2)	0(2)	-1(2)
O12	35(4)	38(4)	28(4)	10(3)	-3(3)	-23(3)
O13	20(3)	46(4)	17(3)	-7(3)	5(2)	-4(3)
O21	15(3)	15(3)	5(3)	-1(2)	-3(2)	3(2)
O22	22(3)	19(3)	15(3)	-1(2)	3(2)	-4(2)
O23	20(3)	42(4)	11(3)	4(3)	-7(2)	4(3)
O31	10(2)	16(2)	15(2)	0(3)	-3(2)	-6(2)
O32	28(3)	27(3)	9(3)	-10(2)	-2(2)	6(2)
O33	19(3)	8(3)	29(3)	3(2)	-4(3)	-2(2)
O41	16(3)	9(2)	14(3)	-3(2)	1(2)	0(2)
O42	18(3)	16(3)	10(3)	-2(2)	2(2)	-3(2)
O43	16(3)	20(3)	20(3)	1(2)	-10(2)	-4(2)
O121	21(3)	24(3)	14(3)	7(2)	-2(2)	-6(3)

O341	8(2)	12(3)	16(3)	-5(2)	-1(2)	3(2)
Re2	10.8(1)	7.9(1)	9.2(1)	-0.1(1)	0.9(1)	0.3(1)
S5	13.2(8)	10.2(8)	9.9(8)	-1.4(7)	4.8(7)	0.4(6)
S6	12.8(8)	9.4(8)	16(1)	1.8(7)	2.0(6)	0.4(6)
S7	12.5(8)	13.7(9)	11.6(9)	-3.6(7)	1.0(7)	1.0(7)
S8	11.3(8)	28(1)	10.4(9)	3.1(8)	-0.7(7)	-2.4(8)
O3	21(3)	11(3)	20(3)	3(2)	3(2)	1(2)
O4	15(3)	19(3)	11(3)	-4(2)	-3(2)	-1(2)
O51	16(3)	9(3)	12(3)	-1(2)	5(2)	-3(2)
O52	15(3)	18(3)	25(3)	-5(2)	10(2)	-2(2)
O53	21(3)	20(3)	11(3)	0(2)	-2(2)	1(2)
O61	10(1)	10(1)	31(2)	3(1)	3(1)	2(1)
O62	10(1)	10(1)	31(2)	3(1)	3(1)	2(1)
O63	10(1)	10(1)	31(2)	3(1)	3(1)	2(1)
O71	13(3)	12(2)	11(3)	-1.6(19)	1.4(19)	-4(2)
O72	17(3)	31(3)	15(3)	-7(3)	2(2)	0(3)
O73	28(3)	11(3)	26(3)	-6(2)	-6(3)	-1(2)
O81	11(2)	20(3)	10(3)	3(2)	-1(2)	-3(2)
O82	23(3)	59(5)	12(3)	-9(3)	-3(3)	-12(3)
O83	19(3)	28(4)	25(3)	16(3)	8(3)	13(3)
O561	10(1)	10(1)	31(2)	3(1)	3(1)	2(1)
O781	12(3)	22(3)	13(3)	2(2)	-1(2)	-1(2)
N1	12(3)	30(4)	19(4)	-8(3)	-4(3)	-6(3)
N2	14(3)	23(4)	13(3)	-4(3)	-3(2)	-2(3)

Table S9: Experimental bond lengths [Å] for **$(NH_4)[ReO_2(S_2O_7)_2]$**.

**Atom–Atom**	**Length [Å]**
Re1 – O1	1.678(6)
Re1 – O2	1.682(7)
Re1 – O11	2.150(6)
Re1 – O21	1.946(6)
Re1 – O31	2.082(5)
Re1 – O41	1.933(6)
S1 – O11	1.485(6)
S1 – O12	1.412(7)
S1 – O13	1.429(8)
S1 – O121	1.676(7)
S2 – O21	1.547(6)
S2 – O22	1.412(7)
S2 – O23	1.422(7)
S2 – O121	1.590(7)
S3 – O31	1.489(6)
S3 – O32	1.417(6)
S3 – O33	1.411(7)
S3 – O341	1.657(6)
S4 – O41	1.560(6)

S4 – O42	1.413(6)
S4 – O43	1.408(6)
S4 – O341	1.600(6)
Re2 – O3	1.692(6)
Re2 – O4	1.686(6)
Re2 – O51	1.949(6)
Re2 – O61	2.088(6)
Re2 – O71	1.939(6)
Re2 – O81	2.100(6)
S5 – O51	1.551(6)
S5 – O52	1.414(6)
S5 – O53	1.422(6)
S5 – O561	1.592(6)
S6 – O61	1.466(6)
S6 – O62	1.410(7)
S6 – O63	1.421(6)
S6 – O561	1.673(6)
S7 – O71	1.556(6)
S7 – O72	1.417(6)
S7 – O73	1.413(6)
S7 – O781	1.592(6)
S8 – O81	1.497(6)
S8 – O82	1.423(7)
S8 – O83	1.405(7)
S8 – O781	1.662(6)

Table S10: Experimental bond angles [°] for **$(NH_4)[ReO_2(S_2O_7)_2]$**.

Atom–Atom–Atom	Angle [°]
O1–Re1–O2	103.1(3)
O1–Re1–O11	88.1(3)
O1–Re1–O21	98.8(3)
O1–Re1–O31	166.0(3)
O1–Re1–O41	92.9(3)
O2–Re1–O11	168.6(3)
O2–Re1–O21	94.1(3)
O2–Re1–O31	90.8(3)
O2–Re1–O41	99.3(3)
O21–Re1–O11	82.0(2)
O21–Re1–O31	81.0(2)
O31–Re1–O11	78.0(2)
O41–Re1–O11	82.0(2)
O41–Re1–O21	159.8(2)
O41–Re1–O31	83.7(2)
O11–S1–O121	101.8(3)
O12–S1–O11	115.3(4)
O12–S1–O13	118.4(5)

O12–S1–O121	102.8(4)
O13–S1–O11	110.7(4)
O13–S1–O121	105.4(4)
O21–S2–O121	101.6(3)
O22–S2–O21	111.2(4)
O22–S2–O23	121.1(4)
O22–S2–O121	109.7(4)
O23–S2–O21	106.3(4)
O23–S2–O121	105.0(4)
O31–S3–O341	101.5(3)
O32–S3–O31	114.5(4)
O32–S3–O341	101.8(3)
O33–S3–O31	110.2(4)
O33–S3–O32	119.1(4)
O33–S3–O341	107.6(4)
O41–S4–O341	100.6(3)
O42–S4–O41	109.1(3)
O42–S4–O341	109.6(3)
O43–S4–O41	107.9(4)
O43–S4–O42	122.5(4)
O43–S4–O341	104.9(3)
S1–O11–Re1	130.6(4)
S2–O21–Re1	132.3(4)
S3–O31–Re1	141.4(3)
S4–O41–Re1	129.2(4)
S2–O121–S1	119.2(4)
S4–O341–S3	120.0(3)
O3–Re2–O51	92.6(3)
O3–Re2–O61	167.7(3)
O3–Re2–O71	99.6(3)
O3–Re2–O81	88.2(3)
O4–Re2–O3	103.5(3)
O4–Re2–O51	97.4(3)
O4–Re2–O61	87.9(3)
O4–Re2–O71	93.3(3)
O4–Re2–O81	168.2(3)
O51–Re2–O61	81.1(2)
O51–Re2–O81	83.1(2)
O61–Re2–O81	80.5(2)
O71–Re2–O51	161.4(2)
O71–Re2–O61	84.1(2)
O71–Re2–O81	83.4(2)
O51–S5–O561	100.9(3)
O52–S5–O51	107.7(3)
O52–S5–O53	122.3(4)
O52–S5–O561	104.9(4)
O53–S5–O51	109.4(4)

O53–S5–O561	109.5(3)
O61–S6–O561	100.3(3)
O62–S6–O61	111.1(4)
O62–S6–O63	119.7(4)
O62–S6–O561	107.0(3)
O63–S6–O61	114.6(4)
O63–S6–O561	101.3(3)
O71–S7–O781	101.2(3)
O72–S7–O71	107.5(3)
O72–S7–O781	105.5(4)
O73–S7–O71	109.8(4)
O73–S7–O72	121.3(4)
O73–S7–O781	109.6(4)
O81–S8–O781	101.2(3)
O82–S8–O81	110.3(4)
O82–S8–O781	106.4(4)
O83–S8–O81	113.8(4)
O83–S8–O82	120.1(5)
O83–S8–O781	102.5(4)
S5–O51–Re2	129.4(3)
S6–O61–Re2	143.0(4)
S7–O71–Re2	130.8(3)
S8–O81–Re2	138.6(4)
S5–O561–S6	120.2(4)
S7–O781–S8	120.8(4)
H1A–N1–H1B	109.4
H1A–N1–H1C	109.5
H1A–N1–H1D	109.5
H1B–N1–H1C	109.5
H1B–N1–H1D	109.5
H1C–N1–H1D	109.4
H2A–N2–H2B	109.5
H2A–N2–H2C	109.4
H2A–N2–H2D	109.5
H2B–N2–H2C	109.5
H2B–N2–H2D	109.5
H2C–N2–H2D	109.5

C. Powder XRD

The crystalline powder was prepared in a borosilicate glass capillary with an outer diameter of 0.5 mm and glass wall thickness of 0.01 mm. The experimental powder data was measured on a *STOE Stadi P* diffractometer (*STOE & Cie* GmbH, Darmstadt, DE) with Mo-$K_{\alpha1}$ radiation (λ = 70.930 pm) at room temperature. The reflections were collected on a silicon-based *Dectris Mythen 1K* detector (*Dectris Ltd.*, Baden-Daettwil, CH). The data was processed with *WinXPOW* (version 3.12). The Rietveld refinement was performed with Topas, refining the lattice

parameters freely using the Thompson-Cox-Hastings profile function.[5] The background was described with six Chebychev polynomials.

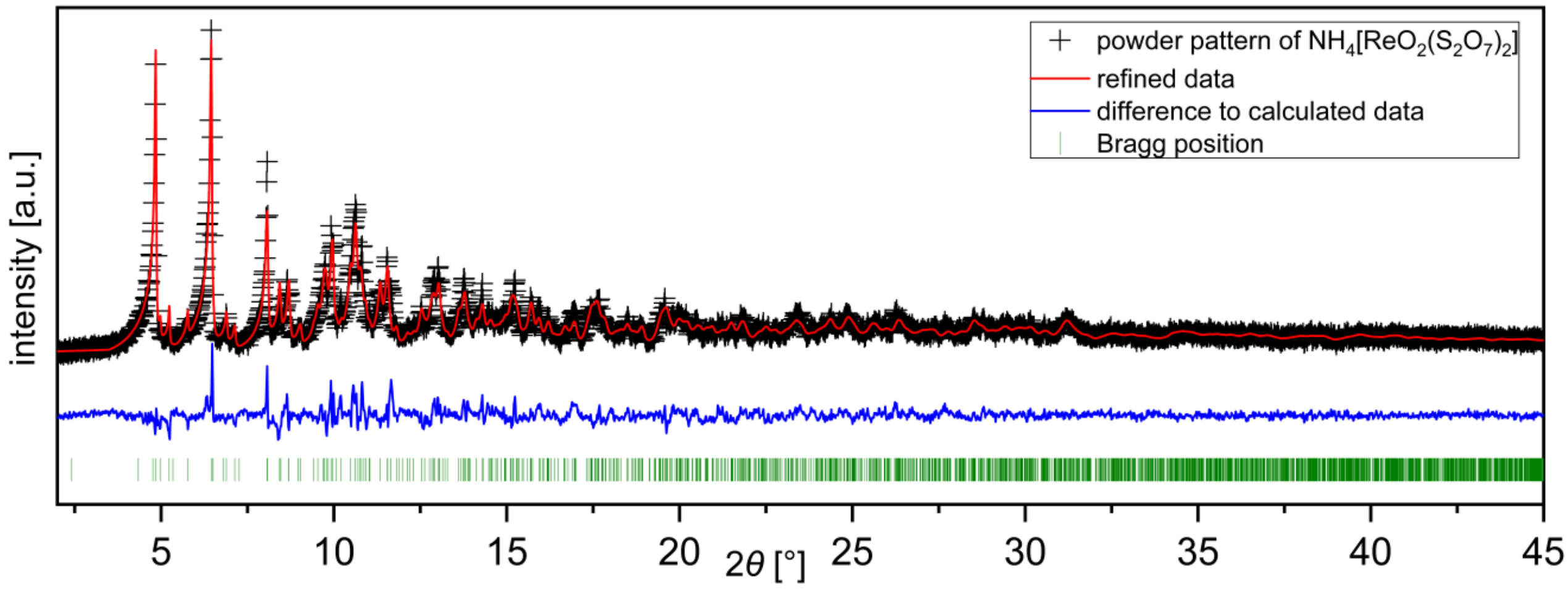

Figure S5: Rietveld refinement plot of **$(NH_4)[ReO_2(S_2O_7)_2]$**. Following profile *R*-factors were received: R_{exp} = 4.29%, R_{wp} = 7.15%, R_p = 5.30%, GOF = 1.66.

D. Vibrational spectroscopy

Raman spectroscopic data were collected with an *inVia Qotor confocal RAMAN microscope* by *Renishaw GmbH* (Pliezhausen, DE) equipped with 10x, 50x and 100x magnification lenses. The spectra were measured on selected single crystals with a green laser (532 nm, 100 mW) with an exposure time of 10000 ms. The data were processed and corrected by using the WiRE 5.1 software. The simulated spectra were simulated according to the geometry-optimized structures (s. chapter **E**).

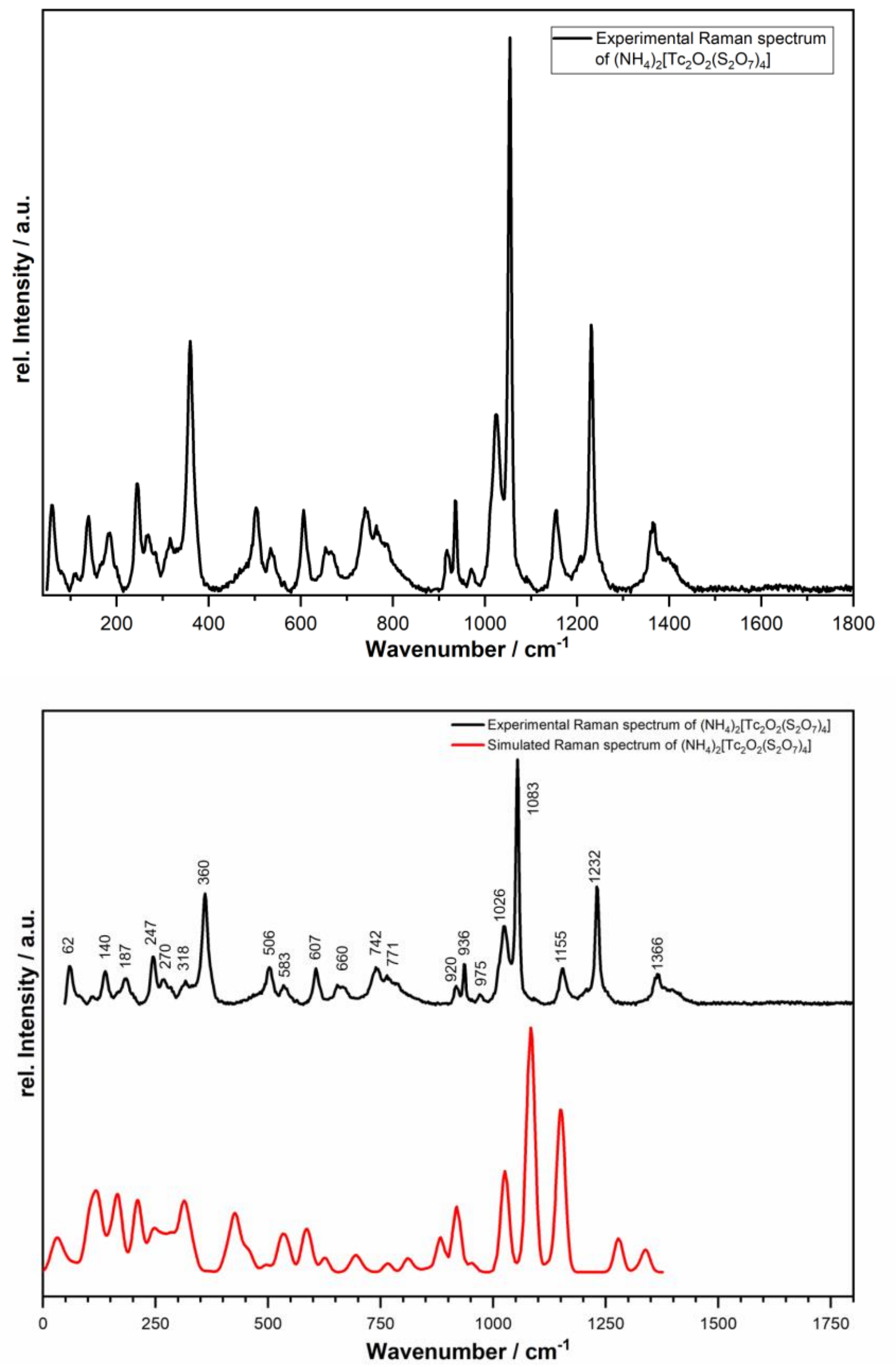

Figure S6: Raman spectrum of **$(NH_4)_2[Tc_2O_2(S_2O_7)_4]$**.

Table S11: Assignment of the experimental and simulated Raman bands (in *italics*) of the anionic **$[Tc_2O_2(S_2O_7)_4]^{2-}$** complex.

Assignment	Raman / cm^{-1}
$\nu_{asym.}$ S–O	1366/ *1337*<br>1232/ *1282*
$\nu_{sym.}$ S–O	1155/ *1147*
$\nu_{sym.}$ Tc–O	1053/ *1083*
$\nu_{asym.}$ Tc–O	1026/ *958*<br>936/ *881*
ν_{sym} Tc–O_b–S	771/ *815*<br>742/ *765*
δ (SO_2-O)	660/ *632*<br>607/ 588

	506/ *541*
Skeletal vibration	360/ *315*
	270/ *250*
	247/ *207*
	187/ *168*
	140/ *122*
	62/ *36*

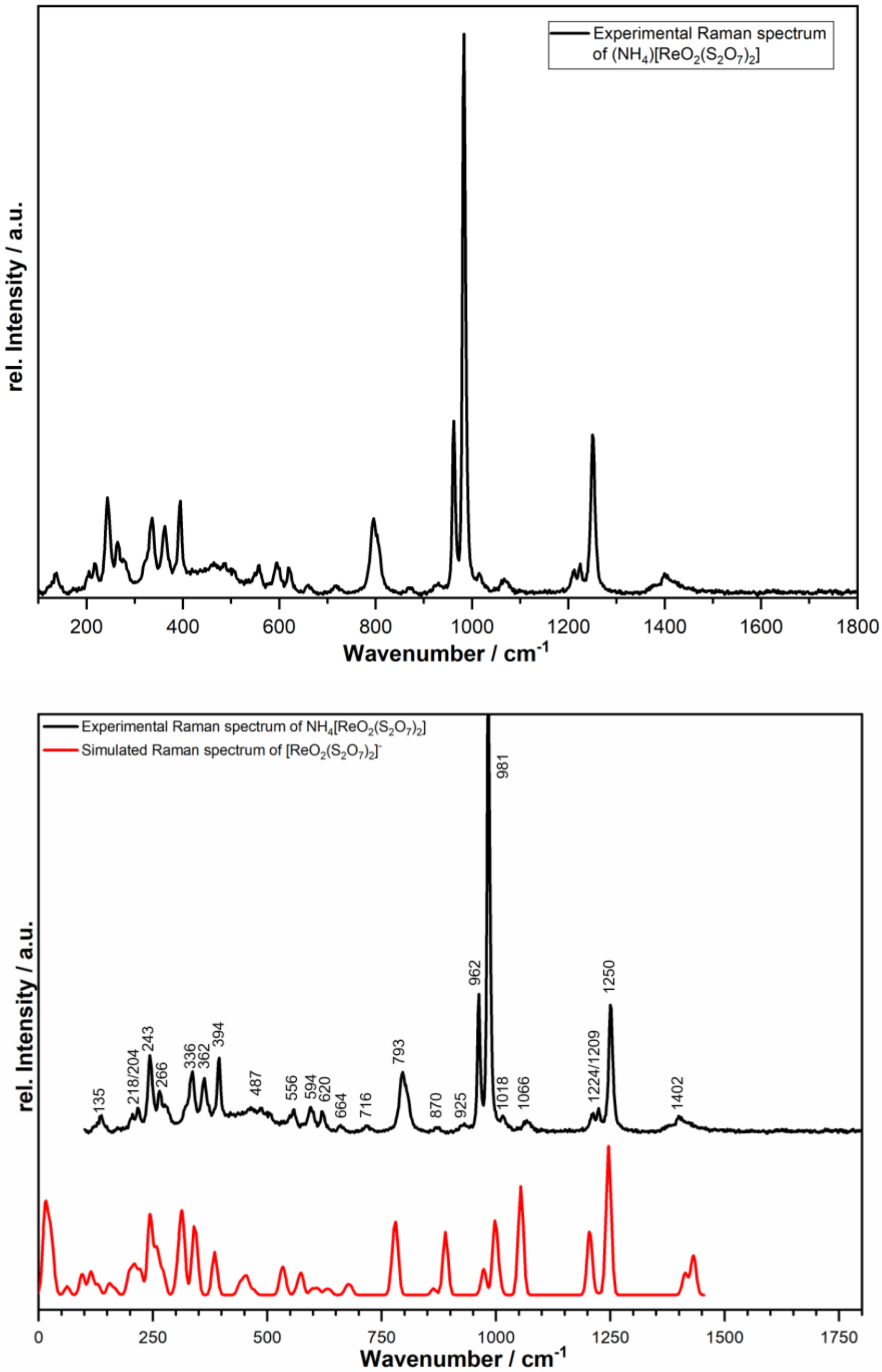

Figure S7: Raman spectrum of **$(NH_4)[ReO_2(S_2O_7)_2]$**.

Table S12: Assignment of the experimental and simulated Raman bands (in *italics*) of the anionic **$[ReO_2(S_2O_7)_2]^-$** complex.

Assignment	Raman / cm^{-1}

$\nu_{asym.}$ S–O	1402/ *1411*, *1433*
$\nu_{sym.}$ S–O	1250/ *1245*
	1224, 1209/ *1206*
$\nu_{sym.}$ Re–O	1066/ *1054*
$\nu_{asym.}$ Re–O	1018, 981/ *997*
	962/ *973*
ν Re–O_b–S	870/ *863*
ν S–O_b–S	793/ *781*
	716/ *670*
δ (SO_2-O)	620/ *613*
	594/ 571
	556/ *531*
Skeletal vibration	394/ *384*
	362/ *344*
	336/ *311*
	266/ *258*
	266/ *242*

F. Results from quantum chemical calculations

Calculation method: Full geometry optimization for $[Tc_2O_2(S_2O_7)_4]^{2-}$ and $[ReO_2(S_2O_7)_2]^{-}$ were performed within density functional theory (DFT) using the TPSSh functional and def2-TZVP basis set for all elements.[6] The calculations were also used for assigning the IR and Raman frequencies. Throughout the study the Orca 5.0.3 program package was used.[7]

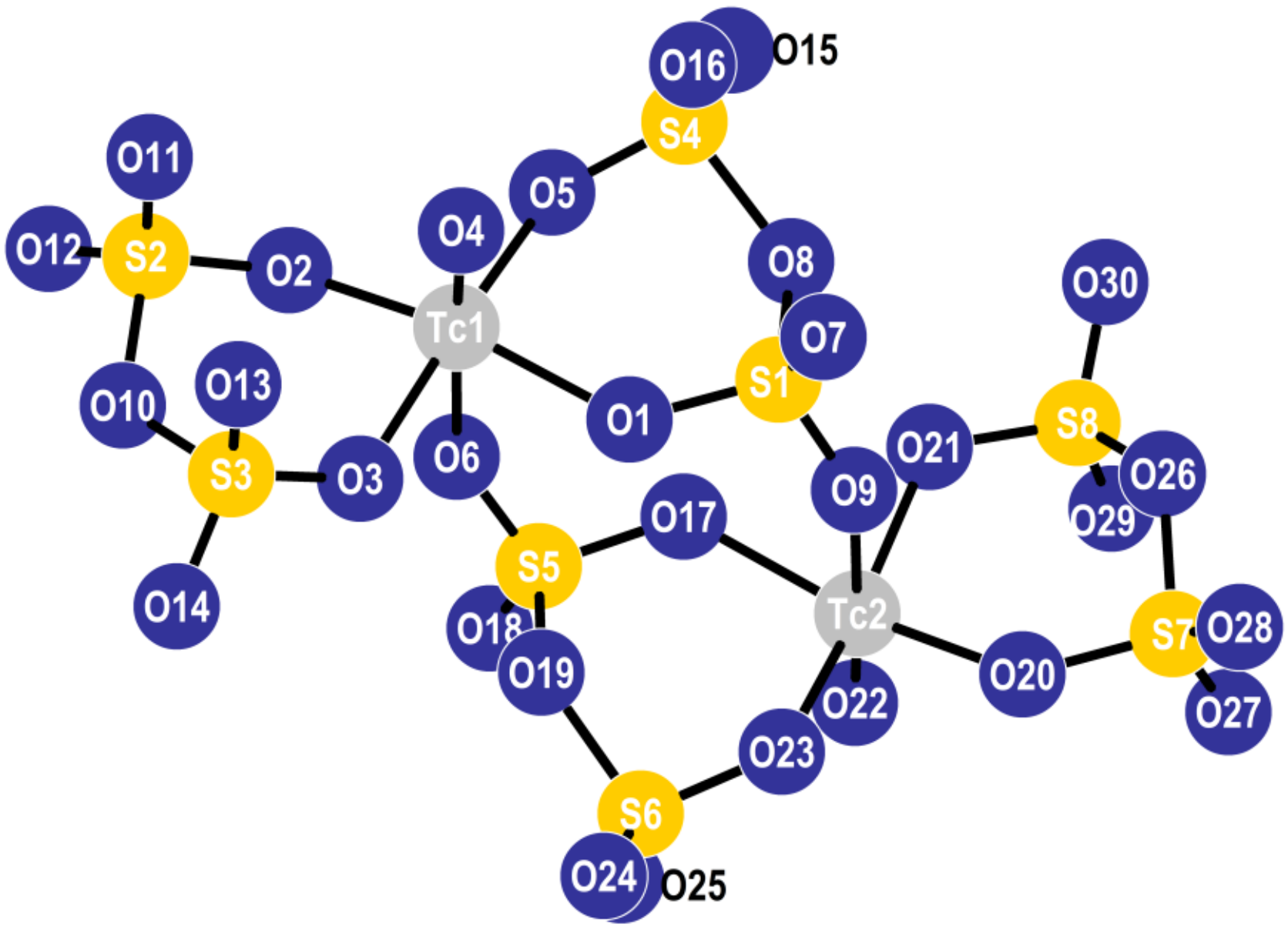

Figure S2: Structure and labelling of the zwitterionic $\mathbf{[Tc_2O_2(S_2O_7)_4]^{2-}}$ of the geometry optimization.

Table S13: Atomic coordinates for the zwitterionic $\mathbf{[Tc_2O_2(S_2O_7)_4]^{2-}}$ from the geometry optimization.

Atom	*x*	*y*	*z*
Tc1	6.26613	2.05532	0.78998
O1	6.61240	0.07536	0.71088
O2	6.32661	3.96702	0.39009
O3	4.76200	1.87368	-0.56362
O4	5.25647	2.07323	2.15856
O5	7.99392	2.24775	1.88103
O6	7.65846	1.97933	-1.02977
S1	7.35180	-0.98760	1.60270
S2	5.13971	5.04595	0.23502
S3	3.45061	2.72877	-0.68122
S4	8.43928	1.38001	3.10059
S5	8.27629	0.91896	-1.88843
O7	6.47235	-1.55154	2.62272
O8	8.62235	-0.19462	2.27228
O9	8.00929	-1.97508	0.68524
O10	4.15505	4.32348	-0.90913

O11	4.41639	5.17858	1.50480
O12	5.67944	6.21938	-0.45336
O13	2.65571	2.76871	0.55098
O14	2.81831	2.50619	-1.98157
O15	9.82328	1.67519	3.46208
O16	7.40174	1.18906	4.11970
O17	9.14476	-0.01956	-0.97876
O18	9.01389	1.39244	-3.05864
O19	6.95748	0.00856	-2.33918
Tc2	9.46100	-2.03585	-1.08484
S6	7.09845	-1.51417	-3.20220
O20	9.30479	-3.97330	-0.69613
O21	10.79695	-1.60935	0.34918
O22	10.47536	-2.04360	-2.37656
O23	7.74220	-2.43846	-2.10374
O24	5.69903	-1.87464	-3.41824
O25	8.01430	-1.29160	-4.32941
S7	10.24011	-4.98128	0.12349
S8	11.89172	-2.60474	0.93248
O26	10.93647	-3.98402	1.31245
O27	11.29435	-5.50287	-0.75631
O28	9.40554	-5.89447	0.90663
O29	12.86256	-2.97629	-0.10669
O30	12.33396	-2.14818	2.24924

Table S14: Bond length (in pm) for the zwitterionic **$[Tc_2O_2(S_2O_7)_4]^{2-}$** from the geometry optimization.

**Atom–Atom**	**Length**
S6 – O25	1.46932
S6 – O24	1.46116
S6 – O19	1.75596
S6 – O23	1.57332
S5 – O18	1.46206
Tc2 – O22	1.64242
S5 – O19	1.66472
Tc2 – O23	2.03827
S3 – O14	1.46296
S5 – O6	1.4978
S5 – O17	1.56925
Tc2 – O17	2.04369
Tc2 – O20	1.98222
Tc2 – O21	2.00576
Tc2 – O9	2.29005
Tc1 – O6	2.29256
S3 – O10	1.7582

S2 – O10	1.67349
S7 – O27	1.46885
S7 – O20	1.60082
S3 – O3	1.56995
S3 – O13	1.4669
Tc1 – O3	2.03166
S2 – O12	1.4636
S8 – O29	1.46985
S7 – O28	1.46415
S7 – O26	1.70091
S2 – O2	1.61148
S2 – O11	1.46736
S8 – O21	1.59046
Tc1 – O2	1.95401
S1 – O9	1.49971
Tc1 – O1	2.01158
S1 – O1	1.57224
Tc1 – O5	2.05248
Tc1 – O4	1.70081
S8 – O26	1.72026
S8 – O30	1.46215
S1 – O8	1.64056
S1 – O7	1.4601
S4 – O5	1.56161
S4 – O8	1.78859
S4 – O15	1.46057
S4 – O16	1.46681

Table S15: Bond angles (in °) for the the zwitterionic $\mathbf{[Tc_2O_2(S_2O_7)_4]^{2-}}$ from the geometry optimization.

**Atom–Atom-Atom**	Angle
O3 – Tc1 – O6	85.2864
O2 – Tc1 – O6	81.4422
O1 – Tc1 – O6	80.3062
O5 – Tc1 – O6	85.0552
O4 – Tc1 – O6	178.3613
O2 – Tc1 – O3	88.5123
O1 – Tc1 – O3	90.7586
O3 – Tc1 – O5	170.3245
O3 – Tc1 – O4	95.5984
O1 – Tc1 – O2	161.7319
O2 – Tc1 – O5	89.4850
O2 – Tc1 – O4	99.9474
O1 – Tc1 – O5	88.1821
O1 – Tc1 – O4	98.2905
O4 – Tc1 – O5	94.0711

Tc1 – O1 – S1	136.3943
Tc1 – O2 – S2	130.6873
Tc1 – O3 – S3	128.2888
Tc1 – O5 – S4	127.0936
Tc1 – O6 – S5	136.8055
O1 – S1 – O9	107.7179
O8 – S1 – O9	103.2041
O7 – S1 – O9	115.9200
O1 – S1 – O8	105.6013
O1 – S1 – O7	111.9701
O7 – S1 – O8	111.5948
O10 – S2 – O12	103.9778
O2 – S2 – O10	102.1287
O10 – S2 – O11	109.9141
O2 – S2 – O12	108.0860
O11 – S2 – O12	121.0858
O2 – S2 – O11	109.8954
O10 – S3 – O14	101.3002
O3 – S3 – O14	110.1658
O13 – S3 – O14	121.1024
O3 – S3 – O10	99.7327
O10 – S3 – O13	107.5361
O3 – S3 – O13	113.8629
O5 – S4 – O8	99.0169
O5 – S4 – O15	110.5623
O5 – S4 – O16	114.4064
O8 – S4 – O15	101.2769
O8 – S4 – O16	106.2331
O15 – S4 – O16	121.6421
O18 – S5 – O19	111.1031
O6 – S5 – O18	115.9561
O17 – S5 – O18	112.2377
O6 – S5 – O19	102.4512
O17 – S5 – O19	105.5643
O6 – S5 – O17	108.6248
S1 – O8 – S4	122.3625
S1 – O9 – Tc2	140.1961
S2 – O10 – S3	122.5946
S5 – O17 – Tc2	130.2104
S5 – O19 – S6	122.9374
O22 – Tc2 – O23	97.2800
O17 – Tc2 – O22	98.1084
O20 – Tc2 – O22	101.4363
O21 – Tc2 – O22	98.7392
O9 – Tc2 – O22	178.2571
O17 – Tc2 – O23	95.1833

O20 – Tc2 – O23	80.7063
O21 – Tc2 – O23	163.9579
O9 – Tc2 – O23	81.7821
O17 – Tc2 – O20	160.3791
O17 – Tc2 – O21	81.7303
O9 – Tc2 – O17	80.5383
O20 – Tc2 – O21	96.8995
O9 – Tc2 – O20	79.8869
O9 – Tc2 – O21	82.1820
O24 – S6 – O25	121.3938
O19 – S6 – O25	107.2014
O23 – S6 – O25	111.6889
O19 – S6 – O24	102.1056
O23 – S6 – O24	110.4976
O19 – S6 – O23	101.4876
Tc2 – O20 – S7	132.0517
Tc2 – O21 – S8	125.9866
Tc2 – O23 – S6	125.3100
O20 – S7 – O27	109.6503
O27 – S7 – O28	120.5315
O26 – S7 – O27	109.4537
O20 – S7 – O28	109.4838
O20 – S7 – O26	103.1746
O26 – S7 – O28	103.0118
O21 – S8 – O29	110.7052
O26 – S8 – O29	108.6786
O29 – S8 – O30	121.0539
O21 – S8 – O26	101.5710
O21 – S8 – O30	110.0629
O26 – S8 – O30	102.6737
S7 – O26 – S8	122.8907

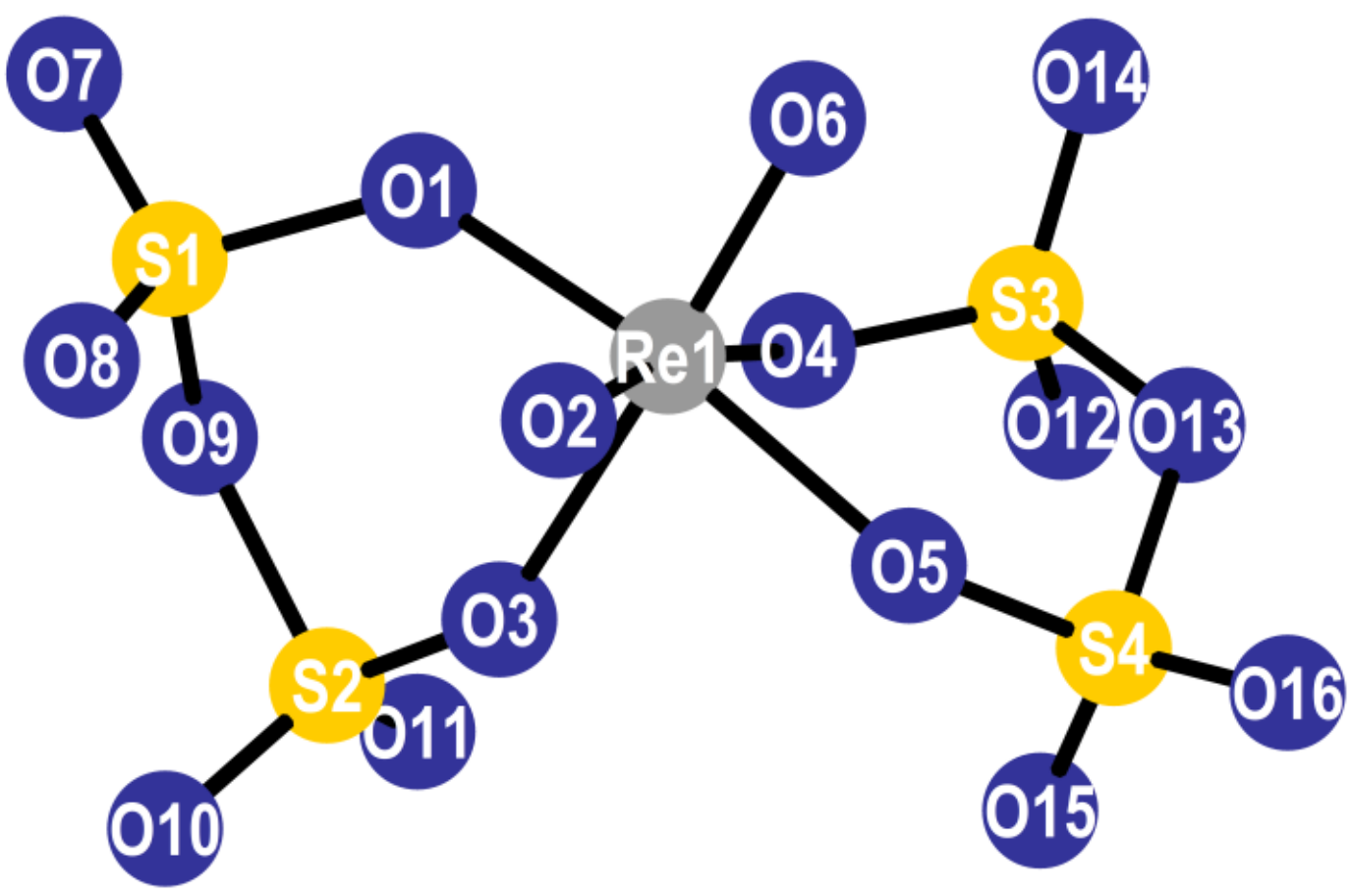

Figure S3: Structure and labelling of the **[ReO$_2$(S$_2$O$_7$)$_2$]$^-$** anion of the geometry optimization.

Table S16: Atomic coordinates for the **$[ReO_2(S_2O_7)_2]^-$** anion from the geometry optimization.

Atom	*x*	*y*	*z*
Re1	8.64893	4.52477	8.74013
O1	10.19054	4.91242	9.86580
O2	7.45856	4.45079	9.94656
O3	8.52525	6.59865	8.71346
O4	9.91406	4.99361	7.11058
O5	7.30318	4.57353	7.34235
O6	9.11474	2.90147	8.59218
S1	10.48773	6.10425	10.85685
S2	9.27770	7.89154	8.94211
S3	10.17611	4.34393	5.77132
S4	7.30413	4.68636	5.76619
O7	11.80460	5.90921	11.36145
O8	9.36914	6.28239	11.72608
O9	10.61005	7.32427	9.83196
O10	8.54302	8.75460	9.80886
O11	9.90373	8.40072	7.76977
O12	10.55227	5.30483	4.78861
O13	8.59592	3.82515	5.38076
O14	10.86702	3.09762	5.86498
O15	7.47069	6.05244	5.39993
O16	6.19653	3.91589	5.30675

Table S17: Bond length (in pm) for the **$[ReO_2(S_2O_7)_2]^-$** anion from the geometry optimization.

Atom–Atom	Length
Re1 – O1	1.94782
Re1 – O2	1.69645
Re1 – O3	2.07773
Re1 – O4	2.11561
Re1 – O5	1.94092
Re1 – O6	1.69529
S1 – O1	1.57828
S1 – O7	1.42366
S1 – O8	1.42777
S2 – O10	1.42685
S2 – O11	1.42323
S2 – O3	1.51328
S2 – O9	1.69964
S3 – O12	1.42497

S3 – O13	1.70841
S3 – O14	1.42808
S3 – O4	1.51141
S4 – O13	1.59968
S4 – O15	1.4241
S4 – O16	1.4253
S4 – O5	1.5802

Table S18: Bond angles (in °) for the **$[ReO_2(S_2O_7)_2]^-$** anion from the geometry optimization.

Atom-Atom-Atom	Angle
O1 – Re1 – O4	85.8564
O1 – Re1 – O5	163.7300
O1 – Re1 – O6	91.3483
O1 – Re1 – O3	81.7136
O1 – Re1 – O2	98.8036
O1 – S1 – O9	100.8478
O1 – S1 – O7	107.0548
O1 – S1 – O8	109.2070
O10 – S2 – O11	120.6945
O12 – S3 – O13	106.9345
O12 – S3 – O14	120.3987
O13 – S3 – O14	101.3889
O13 – S4 – O16	105.0011
O13 – S4 – O15	111.1013
O15 – S4 – O16	121.7714
O2 – Re1 – O4	167.6979
O2 – Re1 – O5	91.5315
O2 – Re1 – O6	102.3041
O2 – Re1 – O3	90.6241
O3 – Re1 – O4	78.7269
O3 – Re1 – O5	85.6652
O3 – Re1 – O6	166.1678
O3 – S2 – O11	113.5732
O3 – S2 – O10	110.6427
O3 – S2 – O9	100.5875
O4 – Re1 – O5	81.6253
O4 – Re1 – O6	88.8923
O4 – S3 – O12	111.5303
O4 – S3 – O13	99.9466
O4 – S3 – O14	113.6349
O5 – Re1 – O6	98.7265
O5 – S4 – O16	106.4069
O5 – S4 – O13	101.6754
O5 – S4 – O15	108.9715
O7 – S1 – O9	105.1355

O7 – S1 – O8	121.7355
O8 – S1 – O9	110.8027
O9 – S2 – O11	101.8795
O9 – S2 – O10	106.7069
Re1 – O1 – S1	131.4687
Re1 – O3 – S2	145.2090
Re1 – O4 – S3	133.7048
Re1 – O5 – S4	136.0297
S1 – O9 – S2	122.0441
S3 – O13 – S4	121.8945

References

[1] Bruker (**2021**). *Apex4*. Bruker AXS Inc., Madison, Wisconsin, USA.
[2] Bruker (**2016**) *SAINT V8.40B*, BrukerAXS Inc., Madison, Wisconsin, USA.
[3] L. Krause, R. Herbst-Irmer, G. M. Sheldrick, D. Stalke, *J. Appl. Crystallogr.* **2015**, *48*, 3–10.
[4] G. M. Sheldrick, *Acta Crystallogr. Sect. A* **2015**, *71*, 3–8.
[5] a) A. A. Coelho, *J. Appl. Crystallogr.* **2018**, *51*, 210-218; b) Topas-Academic (version V6), Brisbane (Australia), **2016**.
[6] T. H. Dunning Jr., *J. Chem. Phys.* **1989**, *90*, 1007–1023.
[7] F. Neese, F. Wennmohs, U. Becker, C. Riplinger, *J. Chem. Phys.* **2020**, *152*, 224108.

Author Contributions

Stefan Sutorius performed the experimental work in course of his PhD work. Julian Heinen did additional work for the analytic measurements. David van Gerven provided the data from quantum chemical calculations. Mathias Wickleder, Jörn Bruns and Erik Strub are the supervisors and leaders of the project.
